# A Meta-Analysis of Microarray Gene Expression in Mouse Stem Cells: Redefining Stemness

**DOI:** 10.1371/journal.pone.0002712

**Published:** 2008-07-16

**Authors:** Yvonne J. K. Edwards, Kevin Bryson, David T. Jones

**Affiliations:** Bioinformatics Group, Department of Computer Science, University College London, London, United Kingdom; University of the Western Cape, South Africa

## Abstract

**Background:**

While much progress has been made in understanding stem cell (SC) function, a complete description of the molecular mechanisms regulating SCs is not yet established. This lack of knowledge is a major barrier holding back the discovery of therapeutic uses of SCs. We investigated the value of a novel meta-analysis of microarray gene expression in mouse SCs to aid the elucidation of regulatory mechanisms common to SCs and particular SC types.

**Methodology/Principal Findings:**

We added value to previously published microarray gene expression data by characterizing the promoter type likely to regulate transcription. Promoters of up-regulated genes in SCs were characterized in terms of alternative promoter (AP) usage and CpG-richness, with the aim of correlating features known to affect transcriptional control with SC function. We found that SCs have a higher proportion of up-regulated genes using CpG-rich promoters compared with the negative controls. Comparing subsets of SC type with the controls a slightly different story unfolds. The differences between the proliferating adult SCs and the embryonic SCs versus the negative controls are statistically significant. Whilst the difference between the quiescent adult SCs compared with the negative controls is not. On examination of AP usage, no difference was observed between SCs and the controls. However, comparing the subsets of SC type with the controls, the quiescent adult SCs are found to up-regulate a larger proportion of genes that have APs compared to the controls and the converse is true for the proliferating adult SCs and the embryonic SCs.

**Conclusions/Significance:**

These findings suggest that looking at features associated with control of transcription is a promising future approach for characterizing “stemness” and that further investigations of stemness could benefit from separate considerations of different SC states. For example, “proliferating-stemness” is shown here, in terms of promoter usage, to be distinct from “quiescent-stemness”.

## Introduction

Stem cells (SCs) have extensive self-renewal capacity and can differentiate into a wide variety of cell types. These are the two defining properties that distinguish SCs from fully differentiated cells. Also central to the study of SCs is the concept of “stemness”, a term coined by biologists to refer to the common genes and mechanisms regulating SC function [1]. Stemness has proved to be an elusive concept to define in terms of individual genes and this has been attributed to the differences in experimental conditions such as the starting SC population and purity [2], [3]. Given that SCs share similar properties, it still remains an attractive proposition to search for the common biological themes and regulatory mechanisms controlling SC function. Whilst much progress has been made to understand the molecular basis of SC function, the description of the molecular control mechanisms common to SCs and to given SC types is incomplete. These are some of the bottle necks that prevent the use of SCs in the treatment of a wider range of diseases.

Complete information regarding the control of gene expression in SCs is necessary to understand the regulation of self–renewal and differentiation. A large number of experiments have shown that the methylation of promoter CpG-islands and histone modifications have an important role in gene silencing and play a central role to genomic imprinting [4], [5]. To exemplify the role of CpG-islands in the control of mouse embryonic SC gene expression, bivalent domains have been characterized as specific modification patterns comprising larger regions of H3 lysine 27 methylation containing smaller regions of H3 lysine 4 methylation [6]. In the genome these bivalent domains largely correlate with the mammalian conserved non-coding elements, the CpG-islands and the transcription factor genes [6]. Bernstein and co-workers (2006) propose that bivalent domains have a role in silencing genes in embryonic SCs “while keeping them poised for activation”. The methods used include histone methylation experiments and bioinformatics techniques. Whilst the role of these domain features has been characterized in embryonic SCs, very little is known about the adult SCs where few such studies have been carried out [7], [8].

**Figure 1:**
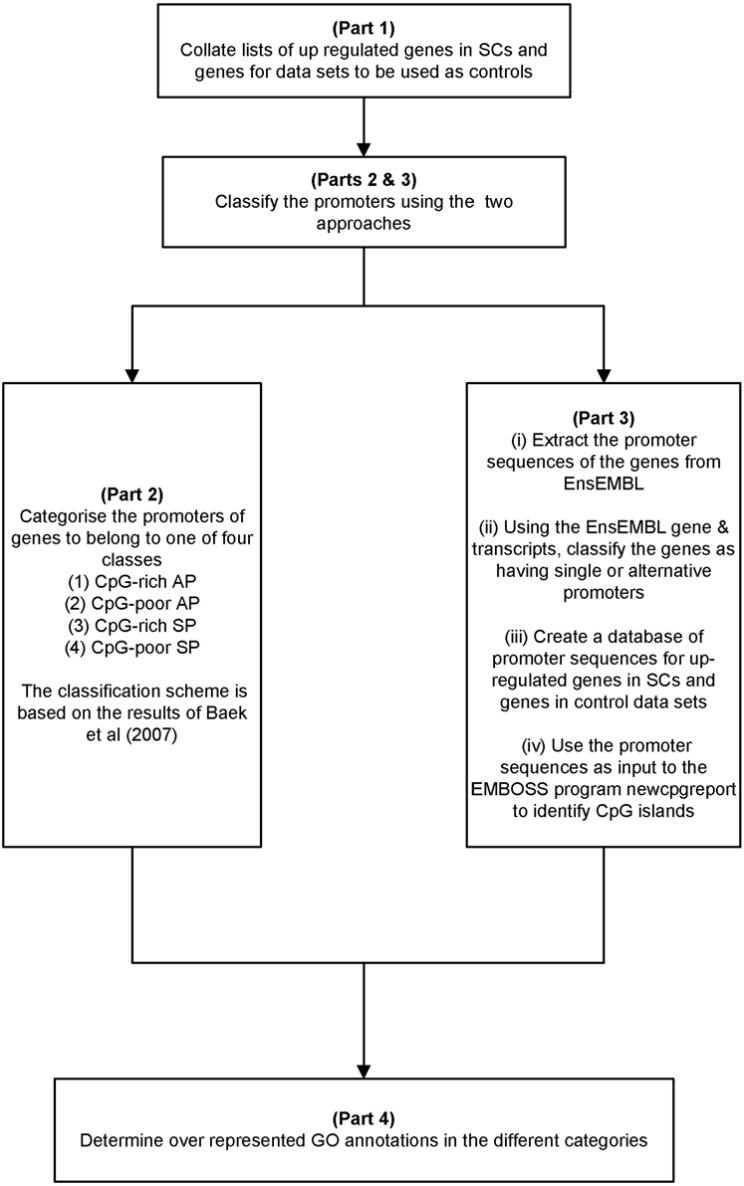
A schematic describing the work flow of the analysis.

Here, a novel meta-analysis of microarray gene expression data to investigate the properties of promoters of up-regulated genes in mouse SCs is described (**Fig 1**). The promoters of genes are characterized in broad terms such as being CpG-rich or CpG-poor and whether the gene is known to have a single promoter (SP) or has alternate promoters (APs). A widely accepted definition of a CpG-island is a genomic region which is longer than 200 bp with high (G+C) content (>50%) and a ratio of observed to expected CpG-dinucleotide greater than that typically found in the genome (>0.6) [9]. The observed versus expected ratio of CpG is normally suppressed in mammalian genome (≈0.1). CpG-islands are in and near approximately 40% of promoters of mammalian genes and with respect to actual frequencies of CpG-islands, the mouse genome contains about 15,500 whilst that of human contains about 27,000 [10]; the mouse genome has about a 40% decrease in the number of CpG-islands compared with the human genome.

The promoter is the genomic sequence located immediately upstream of the transcriptional start site and is defined by the 5′ end of an mRNA transcript. It is a regulatory region that binds the transacting factors required to control gene transcription. A gene has APs if it has multiple transcripts that differ in their 5′ termini [11], [12]. Recent studies estimate that the percentages of genes controlled by APs in mouse and human are 28% and 52% respectively [13]. It is widely accepted that APs are used to drive tissue-specific gene expression, gene expression in development and differentiation processes. AP usage contributes also to the complexity of the mammalian proteome through the generation of different proteins [11]–[15]. An understanding of the SC transcriptome is an important step to understanding the mechanisms regulating biological properties of SCs. Studies that examine the promoters of active genes in SCs are sparse in the scientific literature. There are very few scientific research papers investigating the AP usage in SCs. One relevant and recent study based on ChIP-chip analyses of promoters compared mouse embryonic SCs with fully differentiated tissue and estimated that 28% of genes with RNA polymerase II binding utilize APs [16].

One of the aims of this meta-analysis is to quantify the promoter CpG-richness and AP usage of up-regulated genes in embryonic SCs and establish if it is similar to that of adult SCs. The SCs included in this study originate from different sources (eg., embryonic SCs, neural SCs, retinal SCs, haematopoetic SCs and satellite SCs). Quiescent adult SCs and proliferating adult SCs are included. The microarray gene expression data generated from five independent research groups are reviewed (**Table 1**). This study focuses on the up-regulated genes in mouse SCs and not human SCs due to differences in the number of CpG-islands between human and mouse [10], the significant species differences that occur in the AP usage between mouse and human [13] and the absence of equivalent human SC molecular profiling studies.

**Table 1:** The up-regulated gene lists from the 18 different SC microarray experiments included in the meta-analysis.

Data set	Description	Number of Genes	Reference, species and microarray chip
Ramalhosantos_ESC	ESCs	1788	Ramalho Santos et al 2002 [17] (mouse; mgu74av2)
Ramalhosantos_NSC	Adult NSCs	2458	
Ramalhosantos_HSC	Adult HSCs	1977	
Fortunel_ESC	ESCs	1687	Fortunel et al 2003 [3] (mouse; mgu74av2)
Fortunel_NPC	Adult NPCs	1737	
Fortunel_RPC	Adult RPCs	2230	
Ivanova_ESC	ESCs	757	Ivanova et al 2002 [18] (mouse; mgu74av2)
Ivanova_NSC	Adult NSCs	830	
Ivanova_HSC	Adult HSCs	908	
Venezia_adult_liver_HSC	Adult liver HSCs	955	Venezia et al 2004 [19] (mouse; mgu74av2)
Venezia_fetal_liver_HSC	Fetal liver HSCs	817	
Venezia_5FU	Genes that change over 5FU time course	1488	
Venezia_pgp	Genes expressed on day (2,3 & 6) after 5FU treatment	680	
Venezia_psig	Gene expressed (Venezia fetal liver∩Venezia_pgp)	338	
Venezia_qgp	Gene expressed on day (0,1,10 & 30) after 5FU treatment	808	
Venezia_qsig	Genes expressed (Venezia adult HSCs∩Venezia_qgp)	298	
Fukada_Satellite_Proliferating	Activated satellite cells in adult skeletal muscle	507	Fukada et al 2007 [20] (mouse; moe430)
Fukada_Satellite_Quiescent	Quiescent satellite cells in adult skeletal muscle	659	

**Figure 2:**
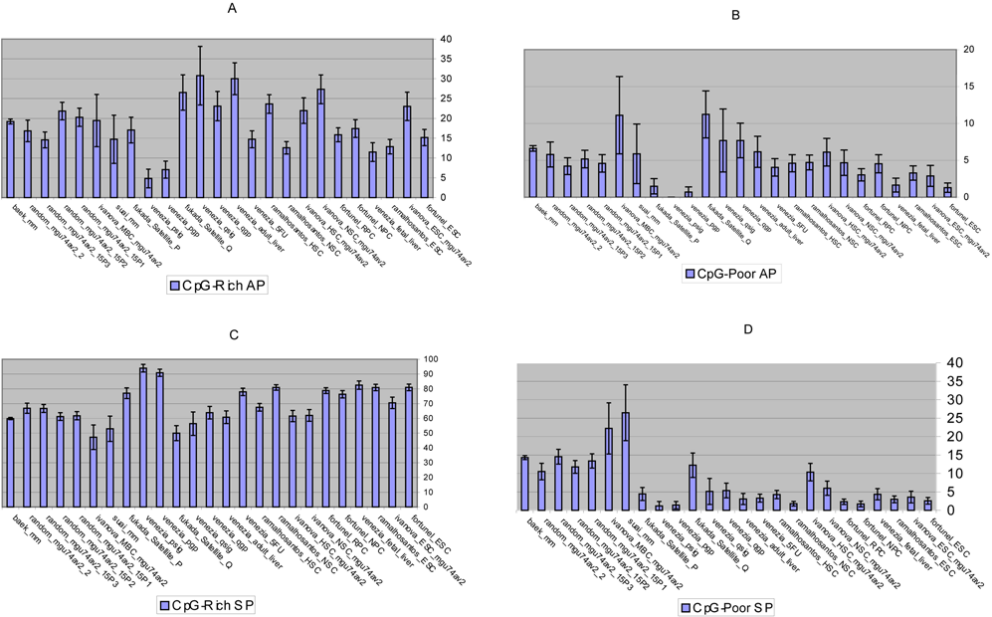
The distribution of genes classed according to the four promoter types (A) CpG-rich AP, (B) CpG-poor AP, (C) CpG-rich SP and (D) CpG-poor SP. The standard error for the percentages are calculated using the equation (p*(100−p)/n)^0.5^ where p is the percentage of genes that belong to a given promoter type and n is the total number of genes classified. The standard errors for the percentages are plotted as error bars. See Fig S1.

The key to abbreviations: SC - stem cell; ESC - embryonic SC; HSC - haematopoietic SC; NSC - neural SC; NPC - neural progenitor cell; RPC - retinal progenitor cell. The pyrimidine analog 5-fluorouracil (5FU) kills cycling HSCs. The spared quiescent HSCs go into cycle after 5FU treatment to re-populate and this is analyzed in the form of a time series [19]. In **Figs 2** **&** **5** venezia_adult_liver is equivalent to venezia_adult_liver_HSC.

## Methods

The methods consist of four parts (**Fig 1**). The first part involves extracting the lists of up-regulated genes in SCs from various microarray studies (**Table 1**). The second part classifies the promoters of up-regulated SC genes to belong to one of four types–CpG-rich AP, CpG-poor AP, CpG-rich SP and CpG-poor SP [as defined in 15]. The third part identifies AP usage of up-regulated SC genes by extracting the promoter sequences from transcripts of genes in the Ensembl database [22], [23] using bioinformatics tools to assess for promoter CpG-richness. The fourth part describes over-represented gene ontology (GO) categories determined for the up-regulated SC genes based on their promoter classification.

### Collecting Lists of Up-regulated Genes in Stem cells

The gene lists collated are up-regulated genes for various types of SCs published by five different research groups (**Table 1**). The details for each gene list were obtained from the supplementary data of the published research. There are 18 data sets classed by cell type or developmental stage (**Table 1**). Three different sets of gene lists are collated as negative controls (**Table 2**). These control data sets are obtained from different analyses. The first set comprises profiled gene expression from 45 mouse samples across a diverse array of tissues, organs and cell lines [21] and also mouse mature blood cells [18]. The second set of gene lists comprises either 10% or 15% of mouse genes from mgu74av2 chip randomly sampled and were derived as part of this analysis. The third set of control gene lists comprises the mouse identifiers in the Baek *et al* (2007) study [15].

**Table 2:** The seven negative control sets included in the meta-analysis.

Data set	Description	Number of genes	Reference, species and microarray chip
Suai_mm	Differentiated tissue–42 tissue types	256	Su et al 2002 [21] (mouse;mgu74a)
Ivanova_MBC_mgu74av2_2	Mature Blood Cells	224	Ivanova et al 2002 [18] (mouse; mgu74av2)
Random_mgu74av2_2	∼10% of chip sampled	1250	Generated in this study (mouse; mgu74av2)
Random_mgu74av2_15P1	∼15% of chip sampled	1900	
Random_mgu74av2_15P2	∼15% of chip sampled	1900	
Random_mgu74av2_15P3	∼15% of chip sampled	1899	
Baek_mm	Transcripts with promoters categorized (mouse)	4189	Baek et al 2007 [15] (mouse;na)

The key to abbreviation: na - not applicable.

### Classifying Promoters of Up-Regulated SC Genes using the Baek *et al* Data

Baek and co-workers (2007) characterized 12,025 promoter regions that are conserved between mouse and human. Of these, 1080 could be reliably assigned as APs and 3109 as SPs. They classed each promoter as “CpG-rich” if the flanking genomic region significantly overlaps one or more CpG-islands or as “CpG-poor” otherwise. According to their classification scheme, the properties of up-regulated SC genes in the four promoter classes (CpG-rich AP, CpG-poor AP, CpG-rich SP and CpG-poor SP) are considered. This section describes the mapping of up-regulated SC genes (**Table 1**) to previously characterized genes and their promoters in mouse and human [15]. The promoters and the corresponding genes (ie., the mouse and human isoforms) are identified by the RefSeq names and the EMBL/DDBJ/GenBank accession numbers [15]. Some mouse isoforms are referenced by the MGI Clone identifiers. To be able to identify common genes between the two different types of data sets, ie., the data from Baek *et al* study [15] and from our study, it was necessary to obtain three types of identifiers (the accession number, the RefSeq name and the MGI Clone identifier) for each mouse probe (**Table 1**). The accession numbers and the RefSeq identifiers were extracted for the probes using Bioconductor [24]. A pipeline was designed to identify the MGI Clone identifier for mouse probes. The pipeline uses two files (MGI_EntrezGene.rpt and MGI_CloneSet_RIKEN.rpt) from the MGI Data and Statistical Reports FTP Site ftp://ftp.informatics.jax.org/pub/reports/index.html. The pipeline included the following three steps. (a) Mapping the probe identifier to the Entrez Gene identifier using Bioconductor functions; Bioconductor version 2.0 and R version 2.5.0 were used. (b) Mapping the Entrez Gene identifier to the MGI Marker identifier. (c) Mapping the MGI Marker identifier to the MGI Clone identifier. These processes are automated with PERL scripts developed in-house. Lastly, a set of java classes was developed to compare the RefSeq identifiers, the accession numbers and the MGI Clone identifiers between the up-regulated SC genes (**Table 1**) and the genes from the Baek *et al* (2007) study [15].

### Classifying Promoters of Up-Regulated SC Genes using Bioinformatics

The entrez gene identifiers of up-regulated SC genes (**Table 1**) were used to extract 1500 bp upstream and 1500 bp downstream of genomic sequence from the transcription start site. The promoter regions were extracted from the Ensembl database using the PERL Ensembl API [22], [23]. The NCBI m37 assembly of the mouse genome was used (Ensembl gene build Oct 2007; database version 48.37a; Ensembl PERL API release 48). A PERL algorithm was developed to define APs. For each gene, the coordinates of the transcription start sites (TSSs) were extracted using the Ensembl PERL API. The TSS coordinates were sorted in ascending order. A genome wide study to identify and characterize APs of human genes was previously carried out using the clustering of capped full length cDNAs and comparing the clusters with the genome sequence and known RefSeq genes. This study provides strong evidence that the true APs are most likely to be separated by at least 500 bp [25]. The APs were identified in this analysis here using a similar definition to previous studies with respect to being at least 500 bp apart from each other [15], [25]. The most 5′ terminal TSS_(i)_ was used as the reference point to search for a downstream TSS_(i+1)_ which passes the filter. If a new TSS_(i+1)_ was identified, it was taken as the updated reference point to search for the next downstream TSS_(i+2)_ and so on until no more could be found. All the promoter sequences were extracted from Ensembl and used as input for an EMBOSS program [26] newcpgreport to report the CpG-rich regions. EMBOSS release 5.0.0 was used. Each gene is described as having a SP or APs. Each gene is described as CpG-rich, CpG-mixed or CpG-less. CpG-rich describe the scenario in which all the promoters for the gene have a CpG-island. A gene is CpG-less when none of the promoters have CpG-islands. A gene belongs to the CpG-mixed category when it has APs and at least one promoter has CpG-islands and at least one promoter has none. PERL programs were written to perform these classifications.

### Over-Represented GO Terms in the Different Categories of Data

Two comparisons were performed. GOstats [27], a Bioconductor package written in R, was used to examine gene ontology (GO) terms [28] that are statistically over-represented in the various data sets (**Tables 1** **,** **2**) and the promoter categories (**Fig 2**). Over-represented GO terms for given data sets were calculated using a classical hyper-geometric statistical comparison against a reference gene list using GOstats. The up-regulated genes expressed in mouse SCs were compared with the genes present on the microarray chip on which the experiment was carried out. The GO term identifiers were included in the study if they are present in the microarray chip with a frequency higher than 8 and if the GO term identifier is present with a frequency equal to or higher than 8 (out of the 24 data sets). Over-represented GO terms for genes that belong to a given promoter class were calculated using a reference gene list. The reference gene list is the member genes of the four promoter classes plus the unclassified genes. The GO term identifiers were included in the study if they are present in the reference gene list and if the GO term identifier is present with a frequency equal to or higher than 6 (out of the 24 data sets). The Baek_mm data set was excluded (**Table 2**). For both comparisons, the entrez gene identifiers were used for gene identification. A PERL script was written to automate this process and perform this analysis.

## Results

### Classifying Promoters of SC Genes using the method outlined in Part 2 (Fig 1)

The genes are categorised to have one of four promoter types according to the Baek *et al* scheme (CpG-rich AP, CpG-poor AP, CpG-rich SP and CpG-poor SP) (**Fig 2** **; Fig S1**). This scheme considers genes and promoter sequences conserved in human and mouse. The key findings are discussed in the following six subsections.

#### (1) In comparison with the controls, the SCs have a higher proportion of genes using the CpG-rich SPs and a lower proportion of genes using the CpG-poor SPs. Comparing the SCs and the controls, the differences in the CpG usage are confined to the SP categories and not the APs

(**i**) The SCs (mean = 72.9%) have a higher proportion of up-regulated genes with CpG-rich SPs compared with the negative controls (mean = 59.5%) (**Table 3** **;** **Fig 2C**). (**ii**) The SCs (mean = 4.2%) have a lower proportion of up-regulated genes with CpG-poor SPs compared with the negative controls (mean = 16.2%) (**Table 3** **;** **Fig 2D**). (**iii**) The SCs (mean = 18.6%) have similar proportions of up-regulated genes with CpG-rich APs compared to the negative controls (mean = 18.1%) (**Table 3** **;** **Fig 2A**). (**iv**) The SCs (mean = 4.2%) have a slightly lower proportion of up-regulated genes with CpG-poor APs compared to the negative controls (mean = 6.2%) (**Table 3** **;** **Fig 2B**). For the two alternate promoter categories (CpG-rich AP and CpG-poor AP), the percentage of genes in the SCs compared to the negative controls differs by less than 2.0%. This difference is small and statistically insignificant. The opposite is true for the SP categories (CpG-rich SP and CpG-poor SP) where the difference between SCs and non SCs is large and statistically significant (**Table 4**).

**Table 3:** The distribution of promoter usage in the SCs and the negative controls (NCs); four promoter categories are shown.

Promoter type	Mean±Standard Deviation		P-value
	SC	NC	
CpG-rich AP	18.62±7.57	18.12±2.81	0.8106
CpG-poor AP	4.21±2.85	6.19±2.32	0.0941
CpG-rich SP	72.93±12.00	59.50±7.16	0.00288
CpG-poor SP	4.24±2.94	16.18±5.88	0.001240

P-values calculated using the Welch two sample t-tests. The promoters are characterized using the Baek *et al* (2007) scheme.

**Table 4:** The promoter usage in the SCs compared with the negative controls.

	AP	SP
CpG-rich	≈	>
CpG-poor	≈	<

#### (2) The embryonic SCs and the proliferating adult SCs use a higher proportion of CpG-rich SPs compared with the controls and the quiescent adult SCs

(**i**) The proliferating adult SCs (mean = 87.3%) have the highest proportion of up-regulated genes using CpG-rich SPs closely followed by the embryonic SCs (mean = 78.7%) and thirdly by the adult SCs (mean = 70.7%). These three SC types use the CpG-rich SPs to a greater extent compared with the quiescent adult SCs and the negative controls (**Table 5** **;** **Fig 2C**). The quiescent adult SCs (mean = 56.8%) and the negative controls (mean = 59.5%) are similar in their use of the CpG-rich SPs. High usage of the CpG-rich SPs is defined as a property of the embryonic SCs and the proliferating adult SCs (**Table 6**). (**ii**) The proliferating adult SCs (mean = 2.4%), the embryonic SCs (mean = 3.4%), the adult SCs (mean = 4.1%) and the quiescent adult SCs (mean = 7.6%) are in consensus with respect to the usage of CpG-poor SPs. All four SC types use this promoter class to a lower extent compared with the negative controls (mean = 16.2%). All SC types are statistically similar with each other and statistically differ from the non SCs (**Table 5** **;** **Fig 2D**). Examining the individual percentages and with the exception of 2 outliers namely, ivanova_HSC_mgu74av2 and fukada_satellite_Q, all the SCs have less than 7% usage of CpG-poor SPs (**Fig 2D**). Low usage of CpG-poor SPs is reported as a property of stemness (**Tables 4** **,** **6**).

**Table 5:** The distribution of promoter usage in the SC types and the negative controls (NCs); the upper right section of the table considers the CpG-rich SPs and the lower left section considers the CpG-poor SPs.

***************	ASC (70.73±8.63)	ESC (78.72±5.53)	PASC (87.29±9.01)	QASC (56.75±6.93)	NC (59.50±7.16)	***************
ASC (4.11±2.89)	***************	0.08415	0.05993	0.04319	0.01649	ASC (70.73±8.63)
ESC (3.39±0.78)	0.5229	***************	0.2384	0.01210	0.001145	ESC (78.72±5.53)
pASC (2.35±1.81)	0.2732	0.4329	***************	0.01123	0.01596	pASC (87.29±9.01)
qASC (7.58±4.03)	0.2714	0.2107	0.1402	***************	0.6	qASC (56.75±6.93)
NC (16.18±5.88)	0.0009633	0.001057	0.0005378	0.03875	***************	NC (59.50±7.16)
***************	ASC (4.11±2.89)	ESC (3.39±0.78)	pASC (2.35±1.81)	qASC (7.58±4.03)	NC (16.18±5.88)	***************

P-values calculated using the Welch two sample t-tests. The promoters are characterized using the Baek *et al* (2007) scheme. The key to the abbreviations: ESC - embryonic SC; ASC - adult SC; qASC - quiescent adult SC; pASC - proliferating adult SC; NC - negative control. The first and the last rows and columns provide the SC type and the mean±the standard deviation are given in parenthesis.

**Table 6:** The promoter usage in the quiescence SCs compared with the proliferating SCs.

	AP	SP
CpG-rich	>>	<<<
CpG-poor	>	≈

#### (3) The quiescent adult SCs up-regulate a higher proportion of genes that have CpG-rich APs compared to the controls and the proliferating adult SCs

(**i**) The quiescent adult SCs (mean = 26.8%) differ in their use of the CpG-rich APs compared with the proliferating adult SCs (mean = 9.6%), the embryonic SCs (mean = 15.6%) and the negative controls (mean = 18.1%) (**Fig 2A** **;** **Tables 6** **,** **7**). (**ii**) The embryonic SCs (mean = 2.3%) and the proliferating adult SCs (mean = 0.7%) are similar in that they use the CpG–poor APs minimally compared with the adult SCs (mean = 4.7%), the quiescent adult SCs (mean = 8.9%) and the negative controls (mean = 6.2%) (**Fig 2B** **;** **Table 7**). The two adult SC states (quiescent and proliferating) are observed to have distinct patterns in promoter usage. With respect to the CpG-rich AP usage, the quiescent adult SCs are similar to the negative controls and both of these differ from the embryonic SCs and the proliferating adult SCs. With respect to the CpG-poor AP usage the converse is true, the proliferating adult SCs and the embryonic SCs are similar to the negative controls and these three differ statistically from the quiescent adult SCs.

**Table 7:** The distribution of promoter usage in the different SC types and the negative controls (NCs); the upper right section of the table shows the CpG-rich APs and the lower left section the CpG-poor APs.

***************	ASC (20.43±6.28)	ESC (15.63±5.16)	PASC (9.63±6.51)	QASC (26.79±3.85)	NC (18.12±2.81)	***************
ASC (4.73±1.02)	***************	0.1989	0.07734	0.08772	0.3703	ASC (20.43±6.28)
ESC (2.27±0.96)	0.005404	***************	0.2630	0.02206	0.4211	ESC (15.63±5.16)
pASC (0.73±0.74)	0.0007444	0.06135	***************	0.02536	0.1438	pASC (9.63±6.51)
qASC (8.87±2.04)	0.06095	0.01845	0.01236	***************	0.03970	qASC (26.79±3.85)
NC (6.19±2.32)	0.1607	0.003795	0.0005364	0.1361	***************	NC (18.12±2.81)
***************	ASC (4.73±1.02)	ESC (2.27±0.96)	pASC (0.73±0.74)	qASC (8.87±2.04)	NC (6.19±2.32)	***************

P-values calculated using the Welch two sample t-tests. The promoters are characterized using the Baek *et al* (2007) scheme. The notes for **Table 5** provide further explanations.

#### (4) CpG-islands are found in the promoters of active genes in SCs more often than in the controls

**Figure 3:**
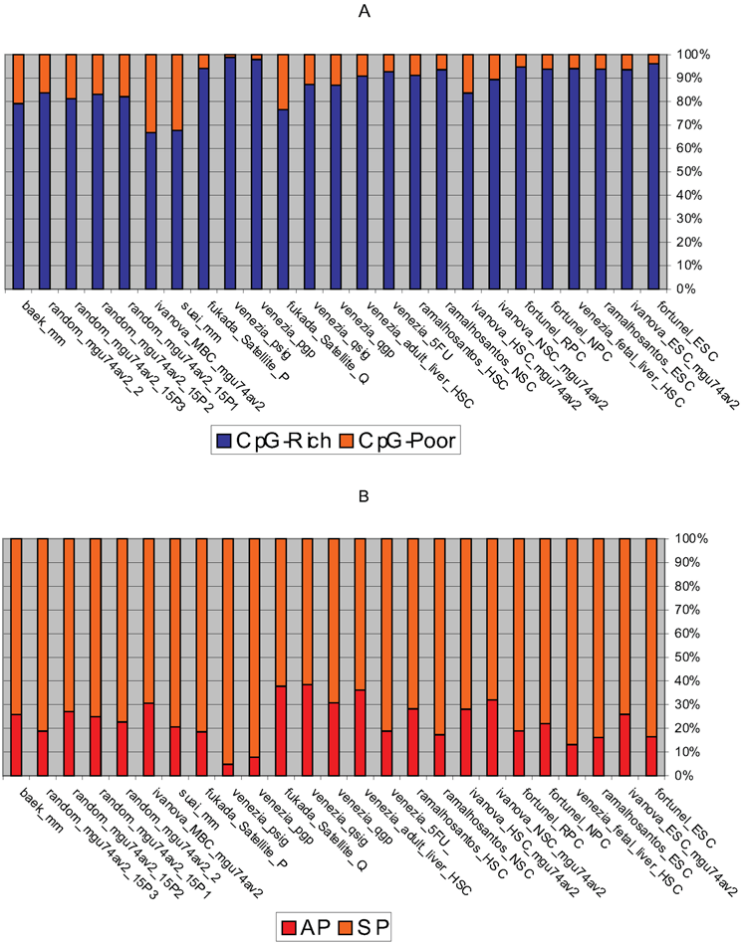
The percentage of the genes classed according to the promoter type. The promoters are characterised based on the Baek *et al* 2007 scheme. Consideration is given to (A) the genes using CpG-rich and CpG-poor promoters and (B) the genes with single and alternative promoters.

The AP and the SP classes are merged so the CpG-rich (CpG-rich SP and CpG-rich AP) and the CpG-poor (CpG-poor SP and CpG-poor AP) classes are considered (**Fig 3A** **;** **Tables 8** **,** **9**). (**i**) The SCs (mean = 91.6%) have a higher proportion of up-regulated genes using the CpG-rich promoters compared with the negative controls (mean = 77.6%). There is a large difference (∼15%) between the two means which is statistically significant (**Table 8** **)**. The proliferating adult SCs (mean = 96.9%), the embryonic SCs (mean = 94.3%), the adult SCs (mean = 91.2%) and the quiescent adult SCs (mean = 83.5%) make higher use of the CpG-rich promoters compared with the negative controls (mean = 77.6%). The first three comparisons show statistically significant differences compared to the negative controls (**Table 9**) and the fourth comparison (ie., the quiescent adult SCs versus the negative controls) is not statistically significant. The proliferating adult SCs (mean = 96.9%) use a higher proportion of up-regulated genes with CpG-rich promoters than the quiescent adult SCs (mean = 83.5%). (**ii**) The adult SCs differ from the embryonic SCs and the proliferating adult SCs by a small value (less than 6%) which is statistically significant (**Table 9**). High usage of CpG-rich promoters is essentially a property of the embryonic SCs and the proliferating adult SCs.

**Table 8:** The distribution of promoter usage in the SCs and the negative controls (NCs); CpG-richness and AP usage are considered.

Promoter type	Mean±Standard Deviation		P-value
	SC	NC	
CpG-rich	91.56±5.37	77.62±7.30	0.001455
AP	22.83±9.92	24.32±4.00	0.5986

P-values calculated using the Welch two sample t-tests. The promoters are classified using the Baek *et al* (2007) scheme. The notes for **Table 5** provide further explanations.

**Table 9:** The distribution of promoter usage in the different SC types and the negative controls (NCs); the upper right section of the table considers CpG-richness, whilst the lower left section considers AP usage.

***************	ASC (91.16±3.55)	ESC (94.35±1.20)	PASC (96.92±2.51)	QASC (83.54±6.08)	NC (77.62±7.30)	***************
ASC (25.16±6.95)	***************	0.04634	0.02793	0.1502	0.001849	ASC (91.16±3.55)
ESC (17.90±5.54)	0.08772	***************	0.2094	0.08717	0.0007531	ESC (94.35±1.20)
pASC (10.36±7.21)	0.04471	0.2126	***************	0.04675	0.0002692	pASC (96.92±2.51)
qASC (35.66±4.25)	0.02222	0.004974	0.01117	***************	0.2462	qASC (83.54±6.08)
NC (24.32±4.00)	0.7759	0.09962	0.06397	0.02046	***************	NC (77.62±7.30)
***************	ASC (25.16±6.95)	ESC (17.90±5.54)	pASC (10.36±7.21)	qASC (35.66±4.25)	NC (24.32±4.00)	***************

P-values calculated using the Welch two sample t-tests. The Baek *et al* (2007) classification scheme is used. The notes for **Table 5** provide further explanations.

#### (5) There is no difference in the AP usage between SCs and the controls

The single (CpG-rich SP and CpG-poor SP) and the alternative (CpG-rich AP and CpG-poor AP) promoter types (**Fig 3B** **;** **Table 8**) are considered. The difference in the percentage of genes using APs in the SCs and the negative controls is small (less than 2.0%) and statistically insignificant (**Tables 4** **,** **8**).

#### (6) The quiescent adult SCs up-regulate a larger proportion of genes that have APs compared with the controls and the proliferating adult SCs

The quiescent adult SCs (mean = 35.7%) have a higher proportion of up-regulated genes using the APs compared to the controls (mean = 24.3%), the proliferating adult SCs (mean = 10.4%) and the embryonic SCs (mean = 17.9%) (**Fig 3B** **;** **Tables 6** **,** **9**). These differences are appreciable and statistically significant. Additionally, in the adult SCs, AP usage is reduced by 10.5% compared with the quiescent adult SCs and increased by 14.8% compared with the proliferating adult SCs. Both these differences are statistically significant.

### Classifying Promoters of SC Genes using the method outlined in Part 3 (Fig 1)

This second study is based on mouse genes and the property of conservation is not given consideration. The promoter sequences of mouse SC genes were extracted from Ensembl and characterised in terms of CpG-islands and APs using the Bioinformatics analysis outlined in part 3 of methods (**Fig 1**).

#### (1) CpG-islands are found in the promoters of active genes in SCs more often than in the controls

**Figure 4:**
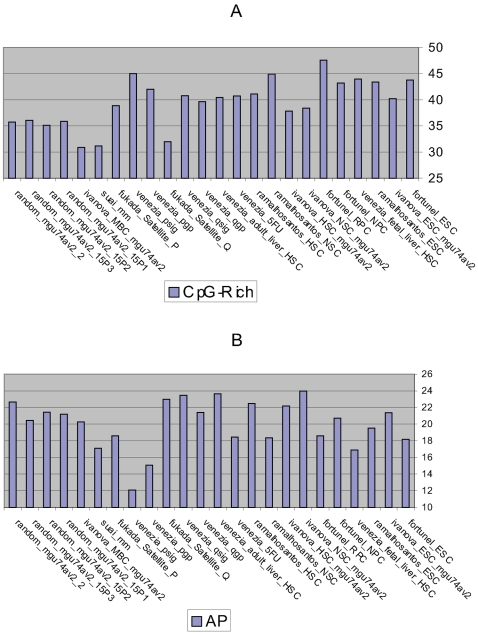
The percentages of the genes classed according to the promoter type. The promoters are characterised using the Bioinformatics methods outlined in part 3 (Fig 1). The y-axis reports the percentage of genes using (A) CpG-rich promoters and (B) APs whilst the x-axis reports the data sets analysed (Tables 1, 2).

The promoters of genes that are CpG-rich and CpG-poor are considered in the context of SCs and non SCs (**Fig 4A** **;** **Tables 10** **,** **11**). The SCs (mean = 41.3%) have a higher proportion of up-regulated genes using the CpG-rich promoters compared with the negative controls (mean = 34.2%). There is approximately 6% difference between the two means and this difference is statistically significant (**Table 10** **)**.

**Table 10:** The distribution of promoter usage; CpG-richness and AP usage are considered to highlight differences between SCs and non SCs.

Promoter type	Mean±Standard Deviation		P-value
	SC	NC	
CpG-rich	41.33±3.48	34.15±2.44	0.0001072
CpG-less	53.52±3.07	59.65±2.61	0.0007695
CpG-mixed	5.15±1.79	6.20±0.68	0.04948
AP	19.89±3.20	20.52±1.88	0.5677

P-values calculated using the Welch two sample t-tests. The promoter classifications rely on the methods described in part 3 (**Fig 1**). The notes for **Table 5** provide further explanations.

**Table 11:** The distribution of the promoter usage in the different SC types and the negative controls (NCs); the upper right section of table considers CpG-richness, whilst the lower left section considers the AP usage.

***************	ASC (41.78±3.29)	ESC (42.84±1.76)	PASC (41.96±3.08)	QASC (37.47±4.78)	NC (34.15±2.44)	***************
ASC (21.05±2.35)	***************	0.4831	0.9351	0.2536	0.0003197	ASC (41.78±3.29)
ESC (19.00±1.92)	0.1471	***************	0.6879	0.1826	0.0001927	ESC (42.84±1.76)
pASC (15.25±3.26)	0.07146	0.1731	***************	0.2542	0.02616	pASC (41.96±3.08)
qASC (22.63±1.08)	0.1668	0.02622	0.04777	***************	0.3527	qASC (37.47±4.78)
NC (20.52±1.88)	0.6467	0.2580	0.09029	0.07264	***************	NC (34.15±2.44)
***************	ASC (21.05±2.35)	ESC (19.00±1.92)	pASC (15.25±3.26)	qASC (22.63±1.08)	NC (20.52±1.88)	***************

P-values calculated using the Welch two sample t-tests. The promoter classifications rely on the Bioinformatics methods described in part 3 (**Fig 1**). The notes for **Table 5** provide further explanations.

#### (2) In terms of CpG-islands in the promoters of genes, the embryonic and the proliferating adult SCs use a higher proportion of genes with these promoter types than the controls whilst the quiescent adult SCs are similar to the controls

One of the aims of the investigation is to quantify the usage of CpG-rich promoters of up-regulated genes in the embryonic SCs and establish if this property is similar to that of the adult SCs. The proliferating adult SCs (mean = 42.0%), the embryonic SCs (mean = 42.8%), the adult SCs (mean = 41.7%) and the quiescent adult SCs (mean = 37.5%) make higher use of the CpG-rich promoters compared with the negative controls (mean = 34.4%). The first three data sets show statistically significant differences compared with the negative controls (**Table 11**). However the fourth SC data set - the quiescent adult SC compared with the negative controls exhibits no statistically significant difference. Whilst statistically significant differences are not observed between the different SC types, statistically significant differences are observed with the SCs versus the negative controls for three out of the four SC types.

#### (3) There is no difference in the AP usage between the SCs and the controls

Each gene is classed as having either a SP or APs and the differences in usage is described for the SCs and non SCs (**Fig 4B** **;** **Table 10**). The difference in the percentage of genes with APs in the SCs and the negative controls is small (less than 1%) and statistically insignificant (**Table 10**).

#### (4) The quiescent adult SCs up-regulate a larger proportion of genes that have APs compared with the proliferating adult SCs

The quiescent adult SCs (mean = 22.6%) have a higher proportion of up-regulated genes using the APs compared with the proliferating adult SCs (mean = 15.2%) and the embryonic SCs (mean = 19.0%). Both these comparisons show statistically significant differences (**Fig 4B** **;** **Table 11**).

### Comparing the results obtained from the two classification schemes

This section compares and contrasts the results obtained from the two classification schemes (parts 2 and 3 of work flow; **Fig 1**). The Baek *et al* classification is based on the promoters and the genes conserved in mouse and human. The second study is based on mouse genes and the property of conservation is not given consideration. When the genes and the promoters are conserved in human and mouse, both SC and non SC populations have double the proportion of genes that are CpG-rich than when conservation is not considered (**Figs 3** **,** **4** **;** **Tables 8** **,** **10**). Considering the differences between the SC and the non SC populations in each study, 14.0% (91.6–77.6%; **Table 8**) and 6.1% (41.3–34.2%; **Table 10**) more SC genes have CpG-islands compared to the non SC populations using the classification scheme of Baek *et al* (2007) and the scheme based on the data extracted from Ensembl and newcpgreport annotations respectively (**Fig 1**). The difference between both SCs and non SC populations again is increased by a factor of 2 when the genes and the promoters are conserved in human and mouse than when the conservation is not considered. Based on the classification scheme of Baek *et al* (2007) and the scheme outlined in part 3 (**Fig 1**) respectively, 1.5% (24.3–22.8%; **Table 8**) and 0.6% (20.5–19.9%; **Table 10**) of genes have APs when comparing SCs to the non SC populations. These are small differences and neither are significant. In both studies, the quiescent adult SCs up-regulate a larger proportion of genes that have APs compared to the proliferating adult SCs (**Tables 9** **,** **11**). Based on the classification scheme of Baek *et al* (2007) and the scheme outlined in part 3 (**Fig 1**) respectively, 15.3% (35.7–10.4%; **Table 9**) and 6.3% (22.6–15.3%; **Table 11**) a larger proportion of genes make use of APs in the quiescent adult SCs compared to the proliferating adult SCs.

The trends obtained from the two classification schemes (parts 2 and 3 of work flow; **Fig 1**) are complementary and supportive. However, the trends are emphasized in the first study compared to the second study. This is most likely due to the conservation of the promoters and the genes in human and mouse in the first study which is not considered in the second study. Conserved non-coding DNA sequences are found near genes involved in early development processes and transcriptional control. The conserved non coding sequences are enriched with regulatory regions such as promoters and enhancers [29]. These regions are enriched with CpG-islands and other regulatory signals for control mechanisms and this observation supports the ethos that conserved genes that have conserved promoters are most likely to share common regulatory features and mechanisms and this is especially true when taking into account what is known about early developmental processes in mammals and also transcriptional control processes [6], [29]. Lastly, the second study shows estimates of CpG-islands and alternate usage of a similar order of magnitude predicted in the mouse genome [10], [13], [16].

### Over-Represented GO Terms in the Different Categories of Data

Over-represented GO terms in up regulated genes in mouse stem cells are examined. Two main studies are carried out. The first takes a bird's eye view of the relationships between over-represented GO terms and various stem cell types. The second study shows associations between over-represented GO terms and the four different promoter types up-regulated in mouse SCs.

### Over-Represented GO Terms associated with SC Types

**Figure 5:**
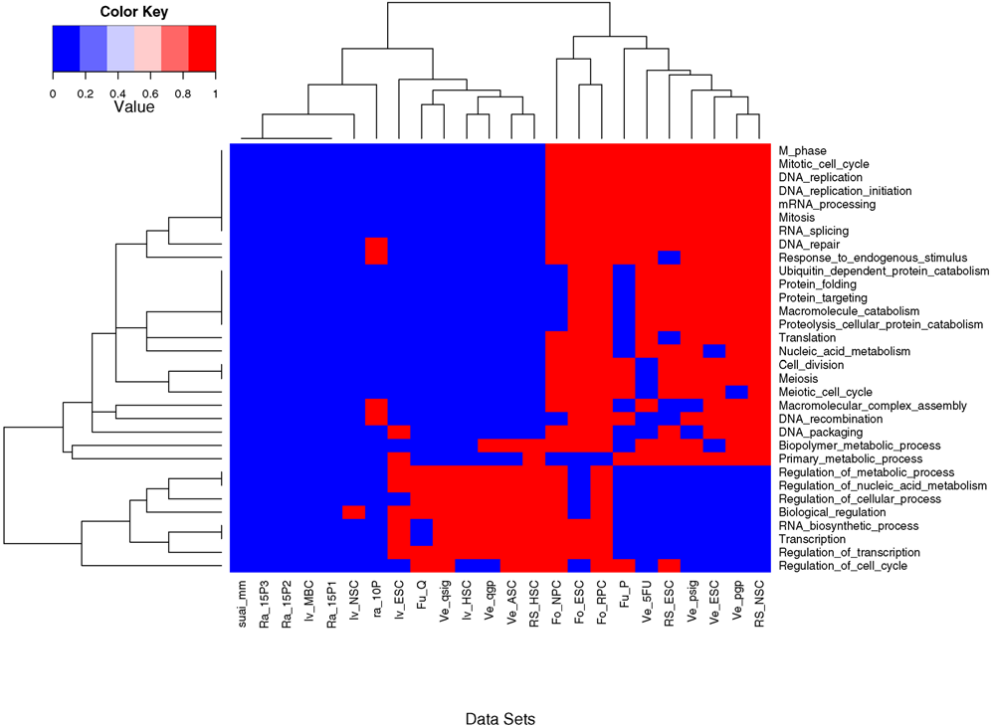
The over-represented GO terms in up-regulated genes of mouse SCs are shown. A heat map showing dendograms clustering the GO terms (x-axis) associated with the different SC data sets (y-axis). The dendogram on the x-axis shows clusters of the various data sets into three main categories. The cluster on the left (x-axis) comprises the negative controls, the cluster in the middle contains the adult SCs and the quiescent adult SCs and the third cluster on the right (of x-axis) largely contains the embryonic and the proliferating adult SCs. The key to the abbreviations follow: RS-Ramalho-Santos; Ve-Venezia; Fo-Fortunel; Iv-Ivanova; Ra-Random_mgu74av2; Fu-Fukada. The GO term identifiers are given in Table 12.

Clustering the up-regulated genes in mouse SCs based on the over-represented GO term identifiers clusters the data into three groups (**Fig 5**; **Table 12**). The first contains the proliferating adult SCs and the embryonic SCs, the second comprises the quiescent adult SCs and the third contains the negative controls (**Fig 5**). There are a few exceptions. For example, the ivanova_ESC data clusters with the quiescent adult SCs and a few adult SCs (proliferating and quiescence status unknown) cluster in one of the three groups. The over-represented GO term identifiers in the embryonic SCs and the proliferating adult SCs are associated with mitosis (the mitotic cell cycle, DNA replication and DNA repair), meiosis (the meiotic cell cycle and DNA recombination), DNA packaging (any process by which DNA and associated proteins are formed into a compact, orderly structure) and the generation of the nucleotide building blocks (**Table 12**). RNA splicing, mRNA processing, translation, protein folding, protein targeting and more general categories are amongst the over-represented terms. The over-represented GO term identifiers in the quiescent adult SCs are associated with regulation: that is the regulation of cell cycle, DNA dependent transcription, metabolic processes and cellular processes. The two over-represented GO terms, not associated with regulation, are transcription and RNA biosynthetic process (**Table 12**). The quiescent adult SCs are enriched with GO terms associated with regulation, whereas the proliferating adult SCs and the embryonic SCs focus on GO terms that are more general in scope. To exemplify, the nucleobase, nucleoside, nucleotide and nucleic acid metabolic process (GO:0006139) is used. This GO term is over-represented in the cluster containing the proliferating adult SCs and the embryonic SCs. Whilst, the regulation of nucleobase, nucleoside, nucleotide and nucleic acid metabolic process (GO:0019219) is over-represented in the quiescent adult SCs. Additionally, this GO term is part of the nucleobase, nucleoside, nucleotide and nucleic acid metabolic process. This suggests that the proliferating adult SCs and the embryonic SCs are associated with the higher order GO terms that include the regulation and suggests that the quiescent adult SCs are focussed on the regulation of this process. Similar analogies exist with the cell cycle GO terms.

**Table 12:** Over-represented GO term identifiers in 24 data sets.

	GO IDs	Description
Over-represented GO terms associated with cluster 1 comprising the embryonic SCs and the proliferating adult SCs	GO:0000279	M phase
	GO:0000278	Mitotic cell cycle
	GO:0006260	DNA replication
	GO: 0006270	DNA replication initiation
	GO:0006397	mRNA processing
	GO:0007067	Mitosis
	GO:0008380	RNA splicing
	GO:0006281	DNA repair
	GO:0009719	Response to endogenous stimulus
	GO:0006511	Ubiquitin-dependent protein catabolic process
	GO:0006457	Protein folding
	GO:0006605	Protein targeting
	GO:0043632	Modification-dependent macromolecule catabolic process
	GO:0051603	Proteolysis involved in cellular protein catabolic process
	GO:0006412	Translation
	GO:0006139	Nucleobase, nucleoside, nucleotide and nucleic acid metabolic process
	GO:0051301	Cell division
	GO:0007126	Meiosis
	GO:0051321	Meiotic cell cycle
	GO:0065003	Macromolecular complex assembly
	GO:0006310	DNA recombination
	GO:0006323	DNA packaging
	GO:0043283	Biopolymer metabolic process
	GO:0044238	Primary metabolic process
Over-represented GO terms associated with cluster 2 containing the quiescent adult SCs	GO:0019222	Regulation of metabolic process
	GO:0019219	Regulation of nucleobase, nucleoside, nucleotide and nucleic acid metabolic process
	GO:0050794	Regulation of cellular process
	GO:0065007	Biological regulation
	GO:0032774	RNA biosynthetic process
	GO:0006350	Transcription
	GO:0006355	Regulation of transcription, DNA-dependent
	GO:0000074	Regulation of cell cycle

The following functional categories are enriched in HSCs, NSCs and ESCs: signalling, transcriptional regulation, DNA repair, cell cycle regulation, cell death, RNA processing, translation, protein folding, ubiquitin pathway, vesicle traffic and toxic stress response [17]. Seven out of the eleven GO terms are over-represented here (**Table 12**) whilst the remaining (4/11) GO terms (signalling, vesicle traffic, cell death and toxic response) are not over-represented (**Table 12**). In the study of Ivanova *et al* (2002), the following functional groups were identified in HSCs (metabolism, RNA binding proteins, apoptosis, protein processing, protein folding, protein synthesis, DNA repair, cell cycle, transporters, cytoskeletal proteins, ECM/cell adhesion, chromatin regulators, transcription factors, intracellular signalling, surface antigens, cell surface receptors and ligands) [18]. Eight out the seventeen GO terms are over-represented here (**Table 12**) whilst the remaining nine GO terms (RNA binding proteins, transporters, cytoskeletal proteins, ECM/adhesion, intracellular signalling, surface antigens, cell surface receptors, apoptosis, ligands), are not over-represented here (**Table 12**). The Ivanova *et al* (2002) study scores functional categories based on counts of genes with the available functional annotation for a single SC type. The present study investigates over-representation using a hyper geometric model and employs a degree of consensus across multiple SC types. The Venezia *et al* study (2004) resulted in the following GO enrichments in HSCs (proliferating; ATP synthesis coupled electron transport, DNA replication, cell cycle check point and hydrogen transport: quiescent; regulation of cell cycle, defense response, protein kinase cascade, cell-cell adhesion) [19]. Four out of these were identified here (**Table 12**). The Fukada *et al* study (2007) resulted in the following GO enrichments in satellite cells (proliferating; synthesis of DNA, RNA, protein and progression of cell cycle: quiescent; regulation of cell growth, TM receptor protein tyrosine phosphatase signalling pathway, cell-cell adhesion) [20]. Whilst all of the proliferating GO enrichments in the Fukada *et al* (2007) study are identified here, none of the quiescent GO terms are identified (**Table 12**). The aim of the meta-analyses here, is to examine commonalities across SCs and SC types. There are more GO terms over-represented that are specific to given data sets in our analysis; many of these are not present at frequencies high enough to pass the filters for consensus (methods). The differences between the enrichment of GO terms in the five independent studies plus this study here can be attributed, in part, to the differences in the methods.

### Over-Represented GO Terms associated with the Four Promoter Types

Comparing the embryonic SCs with the controls, no biological themes are observed for the GO terms over-represented in the CpG-rich APs. In three out of the four embryonic SCs, the two GO terms, the ribonucleoside and the uridine metabolic processes, are over-represented (**Table 13**). Comparing the SCs with the control sets some biological themes are over-represented in the CpG-poor APs and surprisingly three of the GO terms (the positive regulation of B-cell proliferation, the non-apoptotic programmed cell death and the negative regulation of cyclin-dependent protein kinase activity) are common to all the adult neural SCs. Comparing the embryonic SCs with the controls, the GO terms including the mitotic sister chromatid segregation, the M-phase of mitotic cell cycle, the mitotic cell cycle and mitosis are over-represented in the CpG-rich SPs. Themes such as the electron transport and the immune response are over-represented in the CpG-poor SPs (**Table 13**). Associations of GO terms with promoter types were carried out previously [15]. A fair extent of agreement exists between the types of GO terms established previously [15] and those identified here. For example, both investigations identify the GO term immune response with the CpG-poor SPs. The differences observed could be due to differences in methods, eg Baek *et al* (2007) masked genes occurring higher in the list for given promoter types and the predominant use of up-regulated genes in SCs (in this study) compared with their whole genome study.

**Table 13:** Over-represented GO term identifiers in the four promoter categories.

Promoter Type	GO ID	Description	Comments
CpG-rich AP	GO:0019219	Regulation of nucleobase, nucleoside, nucleotide and nucleic acid metabolic process	
	GO:0006350	Transcription	
	GO:0032774	RNA biosynthetic process	
	GO:0006355	Regulation of transcription, DNA-dependent	
	GO:0016481	Negative regulation of transcription	
	GO:0046108	Uridine metabolic process	
	GO:0009119	Ribonucleoside metabolic process	
CpG–poor AP	GO:0030890	Positive regulation of B cell proliferation	In all NSCs
	GO:0016244	Non-apoptotic programmed cell death	In all NSCs
	GO:0043071	Positive regulation of non-apoptotic programmed cell death	In all NSCs
	GO:0045736	Negative regulation of cyclin-dependent protein kinase activity	In all NSCs
	GO:0009411	Response to UV	
	GO:0007050	Cell cycle arrest	
CpG-rich SP	GO:0000070	Mitotic sister chromatid segregation	In all ESCs
	GO:0000278	Mitotic cell cycle	
	GO:0007067	Mitosis	
	GO:0000279	M-phase	
CpG-poor SP	GO:0006954	Inflammatory response	
	GO:0048305	Immunoglobulin secretion	
	GO:0042773	ATP synthesis coupled electron transport	
	GO:0006122	Mitochondrial electron transport, ubiquinol to cytochrome c	

## Discussion

This present work provides new insights regarding the roles of non-coding regions of over-expressed genes in SCs; specifically, of genomic regions in and around gene promoters. We focused our analysis particularly on CpG-islands and on genes that have APs. This is the first large scale survey of CpG-richness and AP usage for up-regulated SC genes. We looked at five independent microarray investigations of mouse SCs. These comprise 25 data sets, over 25,000 genes and over 75,000 promoters. It presents a broad proof of concept that new observations can be obtained from the meta-analysis of published gene lists generated from microarray gene expression experiments. We show that mining of published gene lists obtained from the microarray gene expression can be used to probe underlying patterns of gene regulation. The study uses large volumes of experimental data, and analyses it in ways that have not been previously considered; that is, looking for information in non-coding regions of genes, where previously only the coding regions have been considered [2], [3]. Thus providing new research directions in analyzing microarray gene expression data. However, these data are complex, and there are many challenges in re-analysing the results of these types of experiments at scale. Some of the challenges arise because of variations in how the original experiments were conceived and carried out: that is platform-to-platform [30], lab-to-lab [2], [3] and, of course, species-to-species variability. Other challenges include the lack of standards (e.g. gene naming) for data formats, which make data handling problematic and time-consuming; and critically, important data is sometimes ambiguous or missing from database submissions and supplementary information. This means that authors sometimes need to be contacted to clarify certain issues with regards to the gene lists. In the present work, the impact of platform-to-platform variability was minimized, because much of the data included in the analysis was performed on the mgu74av2 chip (**Tables 1** **,** **2**). We eliminated species-to-species effects by carrying out the entire analysis on mouse SCs. Problems with gene naming were resolved by using the probe identifiers and entrez gene identifiers as the starting point for the analyses, rather than the gene names.

Relatively fewer laboratory–based experiments have been performed on the adult SCs compared with the embryonic SCs, with respect to DNA methylation and chromatin re-modelling [8]. The focus of research efforts has been on the embryonic SCs [16], [31]. CpG-islands are present in the promoters of up-regulated genes of the embryonic SCs to a similar extent of that in the proliferating adult SCs. The up-regulated genes in the embryonic SCs and the adult SCs (with the exception of the quiescent adult SCs) use higher proportions of promoters that are CpG-rich than non-SCs. The results of this investigation suggest that CpG-richness, whether from a proliferating adult SC or an embryonic source, is an important feature for regulating gene expression in SCs. This effect is reduced in the quiescent adult SCs. Our observations suggest that epigenetic regulatory mechanisms such as DNA methylation and/or chromatin re-modelling are important features in the control of gene expression in the embryonic SCs and the proliferating adult SCs and that these effects are used in a different way in the adult quiescent SCs. Stemness has been defined as an unproven notion [1]. This study brings to the fore two related issues. Firstly, considering features associated with control of transcription is a very promising approach for defining stemness as opposed to studying the identity of individual genes. Secondly, in terms of stemness it would appear to be more fruitful to investigate “proliferating-stemness” which is shown here, in terms of promoter usage, to be distinct from “quiescent-stemness”. Our results show different signatures of promoter usage for the SC populations and non-SC populations and among different types of SCs. The results of this research may have possible applications to establish how pure an adult SC population is, ie., what proportion of SCs are in cycle; in quiescent it is widely accepted that about 1–2% of cells are in cycle, whereas in proliferating SCs about 30% of cells are in cycle [reviewed in 19]. Our devised classification system might be usefully applied on a gene chip mRNA expression profile to distinguish whether the cells are SCs or not, and quite importantly whether adult SCs are quiescent or proliferating.

The quiescent adult SCs are shown to express a higher proportion of genes that use APs that are CpG-rich compared with the embryonic SCs, the proliferating adult SCs and the controls. Put another way, with respect to the alternate promoter usage, the proliferating adult SCs and the embryonic SCs are similar to the negative controls, whilst the quiescent adult SCs are different compared to the negative controls. Whilst the proliferating adult SCs and the embryonic SCs have a larger proportion of genes with CpG-rich SPs compared with the controls and the quiescent SCs. Additionally, the adult SCs (ie., pASCs, qASCs and ASCs) collectively display more variability in the use of APs than the embryonic SCs. Usage of the APs versus the SPs in regulating SC function has not been studied widely. Our results suggest that the use of the SPs and the APs differs significantly in the different contexts of the adult SCs. This does make sense in a biological context. It is generally accepted in the scientific literature that the APs are more highly regulated than the SPs [15]. The CpG-rich SP class is the least highly regulated of promoters and linked to house-keeping functions required by most cell types. The other three classes CpG-poor SP, CpG-rich AP and CpG-poor AP fall into classes of highly regulated promoters. In terms of up-regulated genes using CpG-rich SP class, these results suggest that the embryonic SCs and the proliferating adult SCs are more active than the adult quiescent SCs and one of the ways the proliferating adult SCs and the embryonic SCs achieve this mechanistically, is by reducing use of the genes that have APs and increasing the use of genes that have SPs and in particular increasing the use of genes that have CpG-rich SPs.

The overall aim of this research was to gain new insights into the biological properties likely to affect SC gene expression. We designed a novel bioinformatics analysis pipeline and utilized this pipeline to examine previously published gene expression data from SCs in a novel way and to a large extent the overall aim of the meta-analysis was achieved. We were able to provide new insights into the regulation of SC function. This type of meta-analysis in future could rely on the gene expression data deposited in microarray databases such as Array-Express [32] and GEO [33]. Going down this route would allow for further automation to include larger numbers of cell types and species types and will provide for new research directions in analysis of microarray gene expression data.

## Supporting Information

Figure S1 The distribution of genes classed according to the four promoter types: CpG-rich AP, CpG-poor AP, CpG-rich SP and CpG-poor SP. The 100% stacked column compares the percentage value for each promoter type contributing to the total.
(0.72 MB EPS)
